# Pattern Association and Consolidation Emerges from Connectivity Properties between Cortex and Hippocampus

**DOI:** 10.1371/journal.pone.0085016

**Published:** 2014-01-03

**Authors:** Martin Pyka, Sen Cheng

**Affiliations:** 1 Mercator Research Group “Structure of Memory”, Ruhr-University Bochum, Bochum, Germany; 2 Faculty of Psychology, Ruhr-University Bochum, Bochum, Germany; The University of Plymouth, United Kingdom

## Abstract

The basic structure of the cortico-hippocampal system is highly conserved across mammalian species. Comparatively few hippocampal neurons can represent and address a multitude of cortical patterns, establish associations between cortical patterns and consolidate these associations in the cortex. In this study, we investigate how elementary anatomical properties in the cortex-hippocampus loop along with synaptic plasticity contribute to these functions. Specifically, we focus on the high degree of connectivity between cortex and hippocampus leading to converging and diverging forward and backward projections and heterogenous synaptic transmission delays that result from the detached location of the hippocampus and its multiple loops. We found that in a model incorporating these concepts, each cortical pattern can evoke a unique spatio-temporal spiking pattern in hippocampal neurons. This hippocampal response facilitates a reliable disambiguation of learned associations and a bridging of a time interval larger than the time window of spike-timing dependent plasticity in the cortex. Moreover, we found that repeated retrieval of a stored association leads to a compression of the interval between cue presentation and retrieval of the associated pattern from the cortex. Neither a high degree of connectivity nor heterogenous synaptic delays alone is sufficient for this behavior. We conclude that basic anatomical properties between cortex and hippocampus implement mechanisms for representing and consolidating temporal information. Since our model reveals the observed functions for a range of parameters, we suggest that these functions are robust to evolutionary changes consistent with the preserved function of the hippocampal loop across different species.

## Introduction

In 200 million years of mammalian evolution, the anatomy of the hippocampal formation remained remarkable stable given the tremendous changes in brain size and cortical reorganization [1], [2]. The functional role of the hippocampus and its interaction with cortex have been investigated by numerous studies. Cortical regions are regarded as storage for semantic and procedural representations that guide the processing of sensory information (for a review see [3]), reasoning [4], [5] and motor commands [6]–[8]. The hippocampus in turn is a comparatively small region located in the limbic system. Surprisingly, it is able to receive, represent and address cortical information [9], [10]. Moreover, this region is able to quickly generate associations between cortical information [11]–[13]. In particular, these properties make it suitable for spatial navigation and the formation of declarative memories [10], [14]. However, there is relatively little consensus on the neural mechanisms underlying the function of the cortico-hippocampal circuit.

Here we focus on three anatomical properties of the cortico-hippocampal circuit that, to our knowledge, are common to all mammalian species: First, cortical areas have converging connections to the hippocampal formation and diverging back-projections [15]. Since the number of neurons is substantially higher in the cortex than in the hippocampus, either the information represented in the cortex must be compressed to fit in the hippocampus [16] or the hippocampus must use a different coding scheme, e.g., a spatial-temporal code instead of a spatial code. This trivial fact is mostly neglected in computational models. However, since conduction delays between neurons increase linearly with distance [17], a spatially detached subnetwork introduces heterogenous conduction delays, which can be functionally important for the dynamics of the system [18], [19]. Finally, the cortico-hippocampal loop in fact contains multiple, parallel loops [20]. Cortical spikes induce a chain-reaction that can proceed along several pathways, e.g., from parahippocampal areas directly back to the cortex or through hippocampal regions such as the dentate gyrus, CA3 and CA1 [15].

In this study, we investigate whether these anatomical properties are sufficient to account for temporal learning and thereby represent universal design principles, which are exploited in a variety of species-specific implementations. We build a model that consists of a hippocampal input- and output layer attached to a cortex model [18] and test the functional effect of convergence/divergence and heterogenous conduction delays in the connections between the cortical and the hippocampal layers. These *in silico* experiments aim to study the functional role of the cortico-hippocampal circuit without modeling the internal details of the hippocampus. We find that the combination of a high degree of connectivity and synaptic delays permits the network to learn the association between temporally separated patterns. Furthermore, repeated retrieval of stored associations leads to a consolidation of the association from hippocampus to cortex. Since these results are robust against changes in the model parameters, we conclude that these functions are independent of species-specific parameters, making it possible that they are preserved across mammalian species.

## Methods

### The Neural Network

Our simulations are based on the neural network model proposed by Izhikevich [18] for the cortex. In short, it consists of 1000 spiking neurons with 20% inhibitory neurons and 80% excitatory neurons. Each neuron is connected with 10% of the cortical neurons. Inhibitory neurons are only connected with excitatory neurons. The model uses a computationally efficient approximation of the Hodgkin-Huxley neuron [21]. The dynamics of the two state variables, the membrane potential *v* and membrane recovery value *u*, are given by the differential equations



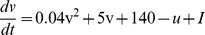
(1)

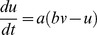
(2)and the auxiliary after-spike resetting



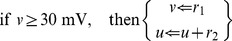
(3)The four parameters in this model can be fit to the spike waveform and bursting behavior of the neuron. Like in the original model, we used a parameter set for regular spiking (*a* = 0.02, *b* = 0.2, *r*_1_ = −65, *r*_2_ = 8 for excitatory neurons; *a* = 0.1, *b* = 0.2, *r*_1_ = −65, *r*_2_ = 2 for inhibitory neurons). Variable *I* is a 1000-dimensional vector representing the input to the neurons (noise and spiking input from other neurons) and is defined as



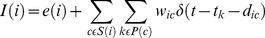
(4)


Each neuron *i* receives random thalamic input *e(i)* (every 10 ms one of the cortical neurons receives a current of 20 mV) and spiking input δ*(x)* from every incoming connection *c* of all post-synaptic connections *S(i)* multiplied by the synaptic weight *w_ic_*. The formula incorporates the time point *t_k_* of all pre-synaptic spikes *P(c)* and the conduction delay *d_ic_*. Conduction delays of the connections can vary between 1–20 ms. Note, that in this notation the variable *w_ic_* denotes the weight of the c-th incoming connection of the i-th neuron, which allows multiple connections between two neurons with differing delays. A MATLAB-implementation of this model is given in the appendix of [18].

**Figure 1:**
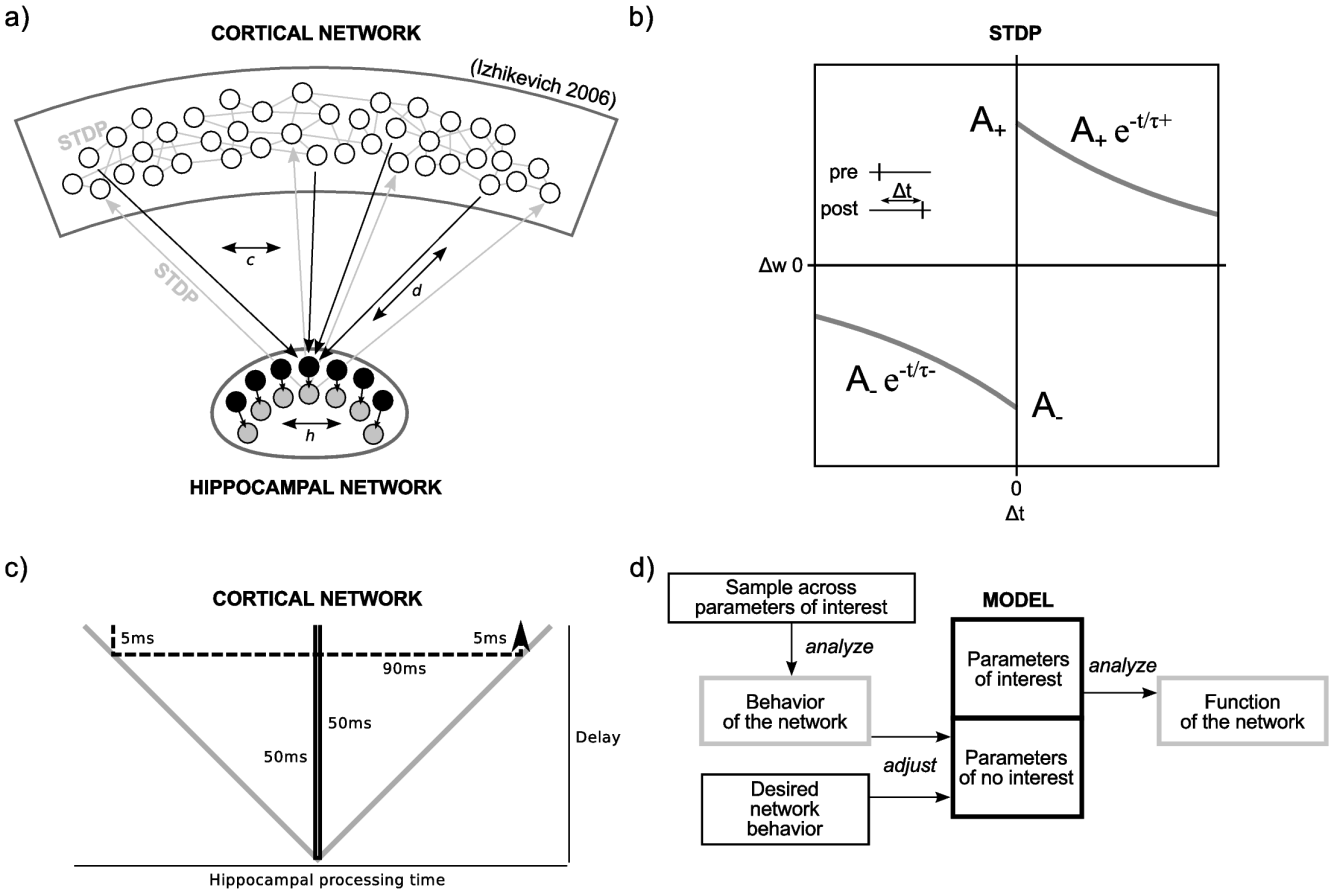
Neural network model and study design. **a**) The network consists of a cortical network as well as a hippocampal input and output layer. It is described by the parameters *c*: number of input/output connections for each hippocampal neuron; *d*: conduction delay between cortical and hippocampal neurons; *h*: number of hippocampal input/output neurons. **b**) STDP rule of the neural network (after Fig. 4 in Izhikevich [18]). **c**) Different interpretations of a conduction delay between cortex and hippocampus. If a signal generated by the cortex needs a time interval of 2*d* to propagate to the hippocampus and back to the cortex, the interval can be represented by two different models. Either there is a delay of *d* between hippocampus and cortex and in the back direction, but no processing takes place in the hippocampus, or the delays amount to only a fraction of *d,* but hippocampal processing consume the remaining time interval. **d**) The design of this study: In a preparatory step, we sample the behavior of the network across the parameters d, h and c and adjust the parameters of no interest to ensure that network behavior is consistent for a range of parameters of interest. Subsequently, we analyze the functional contribution of the parameters of interest (degree of connectivity, delay) on the learning of associations between cortical patterns.

The network is extended by an additional loop representing an hippocampal input and output layer (Fig. 1a). This loop is governed by three parameters. Cortical neurons project to *h* hippocampal input neurons and the same number of hippocampal output neurons project back to the cortex. Anatomical studies suggest that *h* should be considerably smaller than the number of cortical neurons. Each hippocampal input neuron receives *c* connections from randomly selected neurons in the cortex; the same number of random backprojections are made by each hippocampal output neuron. Between hippocampal input and output neurons the connectivity is one-to-one. There is a conduction delay *d* in the connections between cortical and hippocampal neurons. This delay is uniform across the network in the first part of our study, and heterogeneous in the later part. Conceptually, the hippocampal loop differs from the cortical network as only the cortical network can be externally stimulated. Accordingly, only spikes of cortical neurons represent the symbolic activation of an arbitrary information. Hippocampal neurons in turn receive, transform and project back these information.

Spike-timing dependent plasticity (STDP) is defined according to previous work Izhikevich [18] as 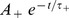 when the post-synaptic neuron fires after the pre-synaptic neuron plus its conduction delay and 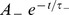 for the reversed case (Fig. 1b). The other parameters are defined as 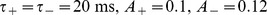. STDP is applied to all connections from excitatory cortical neurons to excitatory and to inhibitory cortical neurons as well as from all hippocampal output neurons to excitatory cortical neurons. These backprojections have been suggested to be the substrate of associative learning [22]. Connection weights can range between 0–10 mV. The simulation results reported in this study were conducted using an additive STDP rule to conform to the work by Izhikevich [18]. However, we also tested the model behavior using a weight-dependent STDP rule that linearly scales the magnitude of STDP by the distance to the upper and lower boundary of the weight [23]. The results were qualitatively identical.

The conduction delay between cortex and hippocampal loop can be attributed either to the spatial distance between cortex and hippocampal loop alone, to hippocampal processing time, or a combination of both. In the first interpretation, spikes generated by the cortex need an interval *d* to reach the hippocampal loop. If there is no internal processing they are projected back to the cortex with the same delay, thus result in a total conduction delay of 2*d*. However, all or some of these delay intervals could be spend on hippocampal processing while the remainder is attributed to synaptic delays (Fig. 1c). We note that none of our current results depends on the particular choice of implementation.

### Assessing Model Behavior

Conceptually, we distinguish between parameters of interest and parameters of no interest. Parameters of interest in this study are the degree of connectivity and the conduction delay between cortex and hippocampal loop as we seek to understand their functional relevance for learning. However, there are many parameters of no interest which can confound the results. For example, the strength of forward connections between cortex and hippocampal neurons, which does not undergo STDP, needs to be determined, and the maximum strength of synapses can be adjusted. In the following, we explain the rationale for adjusting these parameters and reveal their influence on network behavior and function. Figure 1d illustrates the procedure.

Each instance of the model should fullfil basic requirements with regard to the network activity. First, simultaneous activity of 50 cortical neurons (which represent an input pattern) should not cause an infinite reaction chain or oscillatory activity within the cortico-hippocampal network. Second, activity in the hippocampal output layer should be able to evoke spiking in the cortex in a polychronous manner (e.g. precise spike-timing is necessary for cortical spiking).

To confirm that the model behavior is homogenous across the parameters of interest, we generate 3000 networks by sampling across the parameter space (*h*: 10–200, *c*: 1–350, *d*: 20–200 ms) using standard settings as given in [18]. Note, that the parameter *h* is strictly speaking a parameter of no interest. However, as it represents a parameter of the newly introduced hippocampal loop, we include it here to confirm that this parameter is not an important factor for the behavior of the model and therefore treat it as parameter of interest. Like in the original model, each millisecond a randomly selected cortical neuron is stimulated with an input of 20 mV. The networks are simulated for five hours simulation time using an Euler integrator with an integration time step of 0.5 ms. Visual inspection of the spiking behavior in these networks suggested that the addition of the hippocampal loop leads to strong oscillations in the whole network. Therefore, we examined whether the network shows strong oscillations across the entire parameter space. For each simulated network, we determined the number of spiking neurons within intervals of 10 ms for 1 s. Increases between two neighboring time intervals of more than 150 spikes and a spike-frequency between 4–40 Hz (largest coefficient within this range is at least five times greater than the mean of all coefficients obtained from a Fourier transformation of the spiking histogram) indicated strong oscillatory behavior. As the vast majority of models reveals identical behavior, we adjust the parameters of no interest to meet the requirements outlined above. The extensive analysis ensures that the reasons for modifying the parameters of no interest are not specific for a certain point in the space of parameters of interest.

### Hippocampal Learning

To understand the functional implications of the hippocampal loop, we study the storage and retrieval of temporal associations. During learning, two patterns (the cue pattern and the target pattern) are presented to the model 60 times with a given temporal separation. The temporal separation Δ*t* was varied between 10–150 ms. Previous studies investigating networks with heterogenous conduction delays have used polychronous groups (spatio-temporal spike patterns) [18] and “cell assemblies” [24] as functional substrate of cortical information. For our study, we decided to use the latter approach for representing patterns. The main reason for this decision is that preparatory analyses showed that polychronous groups are only reliably reproducible under noise-free conditions with homogenous resting membrane potential and recovery states. Alternatively, additional mechanisms like NMDA receptors [19] which would introduce additional dynamics into the model, have been invoked. In contrast, cell assemblies are widely accepted [25]–[27], allow for a more robust detection of signals and are simpler to implement and interpret. Therefore, in our simulations, patterns are represented by distinct groups of 50 neurons [24]. During presentation through external input all neurons of one group receive a random current (normal distributed around 20 mV, σ = 1 mV).

The number of training trials depends on the strength of STDP. Although it is generally believed that the hippocampus can store information after one trial [28], [29], we decided to use the same STDP rule as in the original model for all connections and instead compensate for the slow learning rate by increasing the number of trials. In the retrieval phase, the network is only stimulated with the cue pattern. The quality of retrieval is quantified by the number of neurons in the target pattern that spike within a 150 ms time window. If not stated differently in the text, we report the average number of spiking neurons after ten retrieval trials.

### Temporal Dispersion

Temporal dispersion roughly reflects the fact that the hippocampal formation has multiple pathways that process information simultaneously, from the recurrent connections within the entorhinal cortex, to the entire hippocampal loop including entorhinal cortex, dentate gyrus, CA3, CA1 and entorhinal cortex. These parallel processing transform timed activity into an extended stream of activity. To investigate how temporal dispersion of cortical signals affects learning through the hippocampal loop, we incorporate this effect in our model. The hippocampal input and output layers are connected with conduction delays uniformly distributed between 10 and 90 ms while the delay between cortex and hippocampal layers is shortened to 1–5 ms. Thus, spikes that were simultaneously generated in the cortex give rise to a stream of activity that reach the cortex, after passing through the hippocampal loop, in a time window of 12–100 ms after spike initiation.

## Results

### Assessing Model Behavior

**Figure 2:**
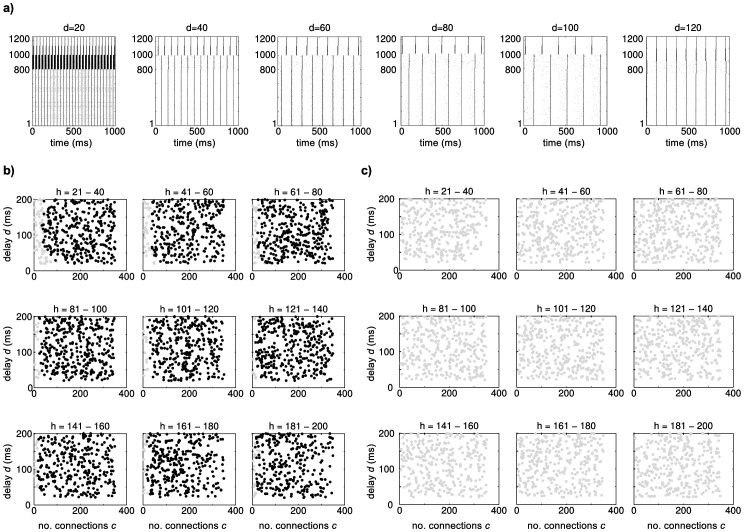
Behavior of the cortico-hippocampal network. **a**) Spiking of networks with parameters *h* = 100, *c* = 300 and *d* between 20 and 120 ms. All other parameters of the model as in Izhikevich (2006). Strong oscillations can be observed with synchronous spiking of all cortical neurons. **b**) Scatter plots of networks in the 3-dimensional parameter space. Networks represented by black dots show strong oscillatory behavior as depicted in a). Gray dots indicate varied spiking behavior. For essentially all models with *h*>50, addition of the hippocampus to the network leads to overload causing these oscillations. **c**) Network behavior across the parameter space after the parameters of no interest have been adjusted as described in the text.

Three thousand networks were generated from randomly selected parameters *h* (21–180), *c* (5–350) and *d* (20–200 ms) and simulated for five hours. Connections were initialized with the same synaptic weights and model parameters (e.g. level of noise, STDP rule) as given in [18]. In the vast majority of the networks, synchronized spikes in cortical and hippocampal neurons can be observed (Fig. 2a). Plots of this synchronized activity revealed that waves of activity oscillate between cortical and hippocampal neurons back and forth in a frequency that results from the conduction delay between cortex and hippocampus (Fig. 2a). For the vast majority of parameter sets that we tested, the network exhibited strong oscillations (Fig. 2b). Only networks with few hippocampal neurons (*h*<50) and low connectivity (c<50) showed more varied dynamics such as varying oscillations and poission-like spiking, as described before [18]. We conclude that the addition of the hippocampal loop to the network fundamentally changed its behavior uniformly across the parameters space. Therefore, adjustments to the model parameters are required to prevent overloading of the network and to obtain the required network behavior (see Methods).

Biological neural networks have various mechanisms such as synaptic scaling [30], [31] and structural plasticity [32], [33] to maintain a certain level of activity. To compensate for the lack of self-organizing mechanisms in our model and to keep it as simple as possible, we manually adjusted some of the parameters of no interest to prevent the system from overloading. The following parameters of the model were altered: connection weights from cortex to the hippocampal loop were reduced to an average of 1 mV, so that synchronized activity of 50 cortical neurons results in 30–50 spiking neurons in the hippocampal input and output layer. The maximum connection weights from the hippocampus to cortex was restricted to 5 mV to ensure that at least four connections are needed to activate a cortical neuron through the hippocampus. The STDP rule originates from an input-free model and contained an increment of 0.01 to every connection every second. We removed this value as otherwise the network would overload once a critical number of connections becomes too strong. Moreover, noise was reduced from 1000 Hz to 100 Hz, as our network will be also driven by external inputs. As shown in Figure 2c, these adjustments prevent the system from overloading across the entire range of tested parameters.

### Association of Patterns

In the following, we examine a model with 100 hippocampal input and output neurons (*h* = 100), high degree of connectivity (*c* = 300) and conduction delays of *d* = 50 ms. Subsequently, the functional properties of this network are compared with networks that lack either high connectivity or large delays.

#### Learning associations of patterns in the cortico-hippocampal network

**Figure 3:**
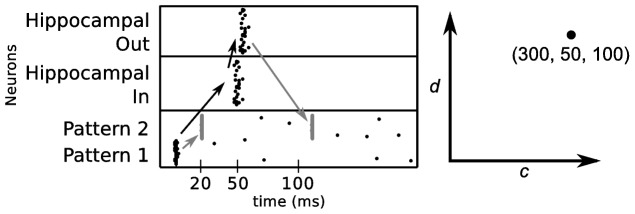
Two cortical patterns can be associated in two different ways. The target pattern (pattern 2) can be activated by the cue (pattern 1) either directly by cortico-cortical connections or indirectly through the hippocampal loop. Low random activity (as shown after 120 ms) do not cause any spiking in the hippocampus. Black arrows show connections which are predefined and are not modified by STDP. Gray arrows indicate the connections that undergo STDP and therefore are involved in learning.

Figure 3 illustrates two different ways, in which an association between a cue and target pattern can be stored in the network. If the temporal separation between the cue and target pattern is short enough, the association can be stored directly in the cortical connections between both groups of neurons via STDP. Additionally, the cortical pattern generates a spatio-temporal signature in the hippocampal loop that can be linked to the cortical target pattern via STDP in the hippocampo-cortical connections. To test for the uniqueness of this spatio-temporal pattern in the hippocampal loop, we created 1000 patterns of 50 randomly selected neurons in the cortex and recorded for each cortical pattern the spiking activity in the hippocampal output layer (activity in the input and output layer is identical). Compared with the recordings of the remaining 999 patterns, we found that no pair of signatures had an overlap of more than 50% (allowing for a tolerance of 2 ms). Thus, a small set of hippocampal neurons can represent a variety of cortical information, through relative spike timing.

To study the storage of associations in our network quantitatively, we defined four different stimuli A, B, C and D, represented by activation of neurons 1–50, 51–100, 101–150 and 151–200. In the learning phase, first stimulus A and B were presented to the network 60 times separated by Δ*t = *120 ms. Subsequently, stimuli C and D were presented under the same conditions. During recall, we presented either stimulus A or C to the network and measured retrieval quality of the patterns B and D. If during presentation of stimulus A, spikes representing pattern B, but not D, occur and vice versa for pattern C, we can conclude that the network is able to disambiguate the two pairings.

**Figure 4:**
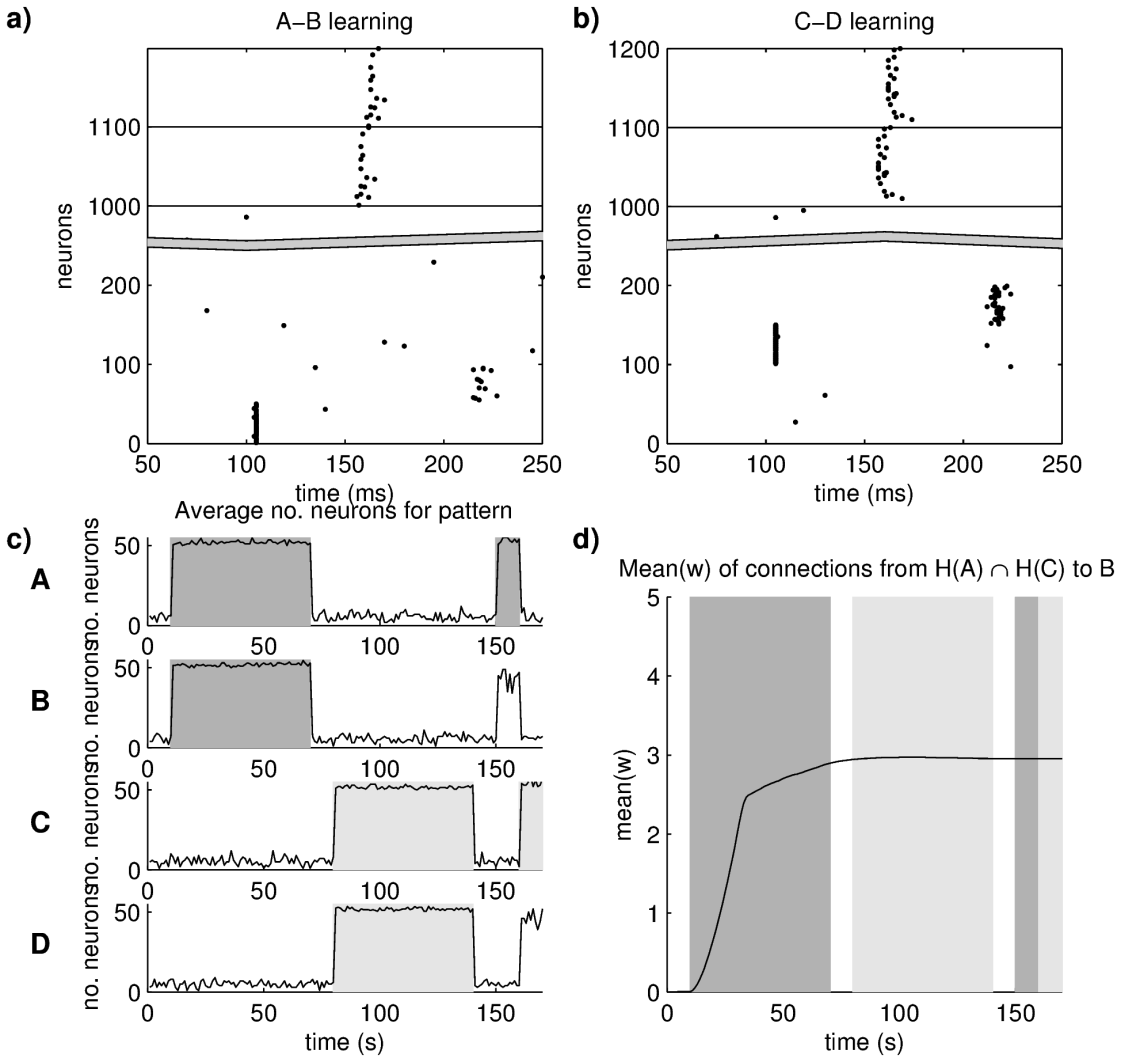
Pattern association through a hippocampal loop. Panel **a**) and **b**) show the recall of two previously learned associations. Both recalls were initiated by an external cue at around 100 ms, which evoked a sparse temporal pattern in the hippocampal layers, which in turn drove the target pattern in the cortex. **c**) Average number of neurons for all four patterns during learning and recall. Grey bars indicate when patterns have been externally stimulated. **d**) Mean weight for connections between hippocampal neurons that spike in response to pattern A and C (H(A) and H(C), respectively) to target patterns B. Grey bars as in c). During the first learning phase, the connection weight increase and thereby contribute to the association of pattern A with B. Although in the second learning phase the same hippocampal neurons are also activated by pattern C, their connections to neurons of pattern B remain stable indicating that the spatio-temporal sequence of hippcampal neurons is important for storing unique associations.

The analysis revealed that pattern A and C produced different spatio-temporal patterns in the hippocampal loop (Fig. 4a and b). Since at least four hippocampal connections with maximal weight are necessary to evoke a spike in a cortical target neuron, both hippocampal patterns were distinct enough to evoke different patterns in the cortex without interference with previously learned associations. Accordingly, the activation of cue pattern A lead to significantly more neurons spiking for pattern B compared to neuron spiking for pattern D and vice versa for cue pattern C (based on a Mann-Whitney-Wilcoxon after 10 repetitions, p<10^−12^ for both cue patterns) (Fig. 4c). Note, that every 10 ms a random neuron receives a current of 20 mV and neurons representing the cue pattern receive a current of 20 mV for 1 ms. Depending on their current state, the spike can emerge with a delay of 4–6 ms. Therefore, noisy spiking occurs in the network. Since some hippocampal neurons spike in response to both patterns A and C, one might suspect that the injection of pattern C during training could retrieve pattern B and thereby introduce an erroneous association between C and B. However, we found that during the second learning phase the connection weights from those hippocampal neurons to B do not increase (Fig. 4d), as their spike-timing evoked by pattern C is too different from their spike-timing evoked by pattern A.

#### Polychronous activation of cortical patterns

**Figure 5:**
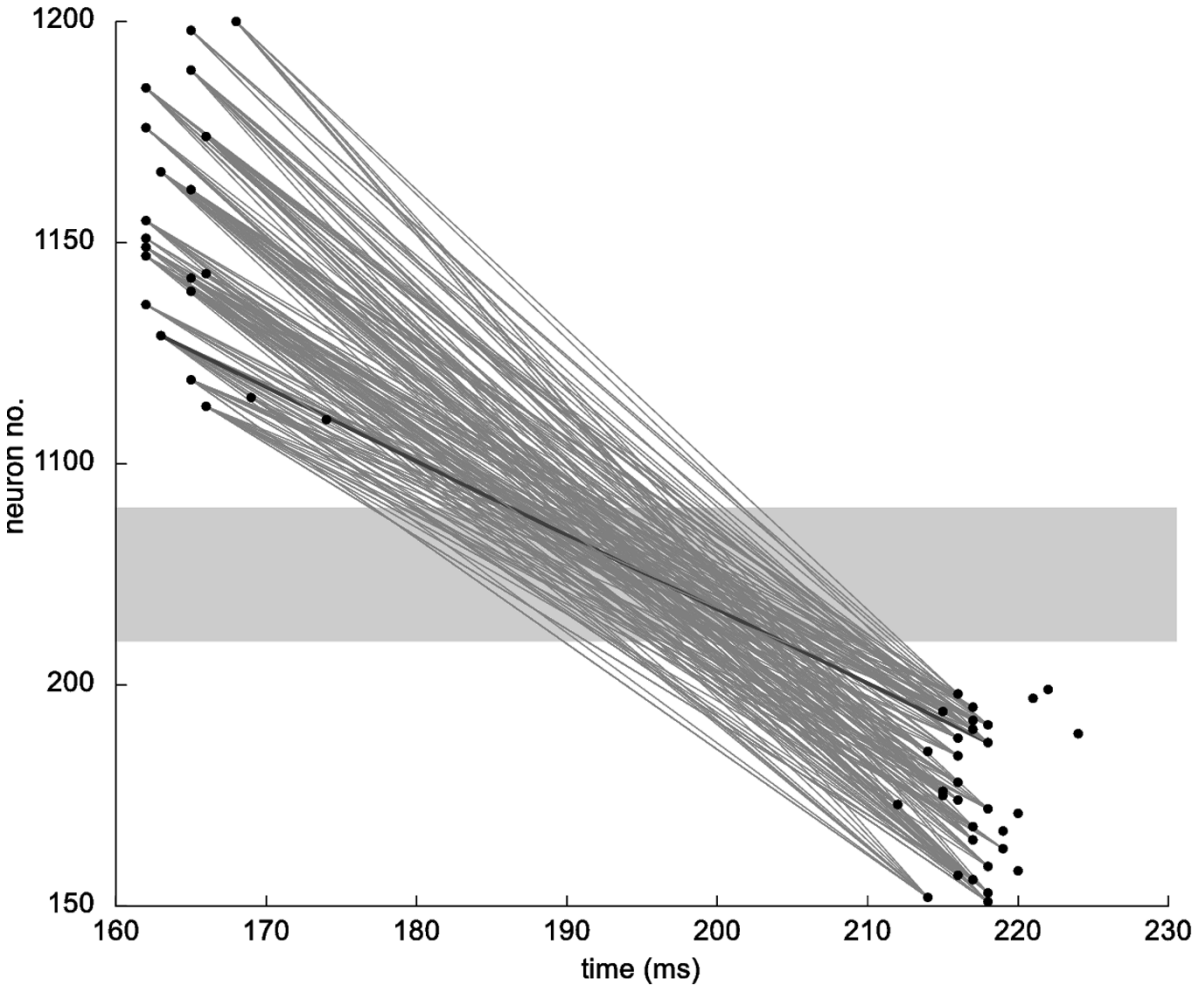
Neurons in the target pattern are driven by three different mechanisms. Thick lines mark connections, where one hippocampal output neuron can generate a spike in a cortical neuron. Thin lines indicate that these connections alone cannot generate a spike. Instead timed activity of more than one hippocampal neuron is needed to elicit a spike in the cortical neuron. Unconnected dots represent cortical neurons whose spiking is not directly evoked by the hippocampus, but by recurrent cortical inputs. Already in the learning process, autoassociative connections between the cortical neurons in target pattern emerge.

Due to the high degree of connectivity between cortex and hippocampus, one might suspect that single hippocampal neurons could directly evoke spiking in the cortical target neurons. For example, a hippocampal output neuron might have multiple connections to a cortical neuron with maximal connection weight. A spike of this neuron could then directly elicit the cortical activity pattern and, therefore, the hippocampal neuron would be a “grandmother cell” for the cortical pattern. The alternative coding scheme is a distributed code in which multiple hippocampal neurons together encode a cortical pattern and in which each hippocampal neuron participates in the encoding of multiple cortical patterns [36]. To examine whether distributed spiking in the hippocampus is necessary for addressing cortical patterns in our model, we determined all connections between hippocampal neurons evoked by pattern C and cortical neurons that contributed to spiking of neurons representing pattern D (neurons 151–200). An examination of these connections showed that only one of 21 hippocampal neurons can directly cause a spike in a cortical target neuron as it contains four connections to the target neuron. All remaining hippocampal neurons do not have enough connections to the target neurons to activate them directly. Instead, the right spike-timing of the hippocampal neurons is necessary to activate the target pattern (Fig. 5). Additionally, the analysis reveals that not all cortical neurons of pattern D are directly activated by the hippocampus. Neurons of the recalled pattern activate each other making the recalled pattern stronger.

#### Critical factors for learning associations through the hippocampal loop

To demonstrate that only a network with high connectivity and large delays is able to bridge a large temporal separation, we generated four networks to test all combinations of the parameters connectivity and delay, e.g. low connectivity/low delay, high connectivity/low delay, low connectivity/high delay and high connectivity/high delay. We studied in all four networks for which temporal separations Δ*t* the networks could learn the association between two patterns (neurons from 1–50 and 51–100). The separation between the two patterns was sampled from 10 ms to 200 ms in steps of 5 ms (beyond 200 ms no learning was possible). After 60 learning trials, we determined the retrieval quality (see Methods).

**Figure 6:**
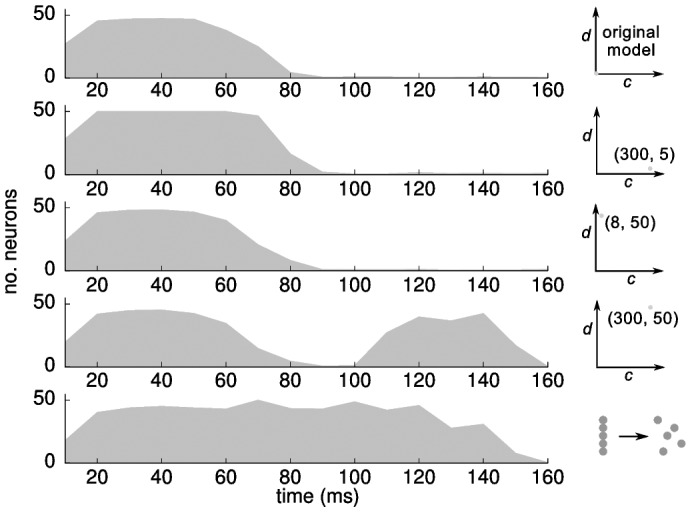
Influence of anatomical properties of the hippocampal loop on the capability to associate patterns across. Each plot depicts the number of target neurons that are activated during recall as a function of the temporal separation between the cue and target patterns during training. From top to bottom: original model by Izhikevich [18], hippocampal loop with high connectivity/low delay, low connectivity/high delay, high connectivity/high delay, and high connectivity/high delay+temporal dispersion.

The analysis revealed that in all models two stimuli can be directly associated with each other when Δ*t <*60–70 ms (Fig. 6). However, only the network with high connectivity and high delay is able to learn an association across a time span that goes beyond the cortical association capabilities. For *d* = 50 ms, the temporal separation between cue and target patterns can range from 110–150 ms (Fig. 6, fourth row). Here, the delay between cue and target pattern does not need to be constant across presentations in the training phase. Even if the delay randomly varies between 110–150 ms, the target pattern becomes associated with the cue pattern with no discernible reduction in the recall quality (data not shown). While this analysis shows a clear benefit of high connectivity and large delays, cortical patterns separated by a time span of ∼70–110 ms cannot be associated with each other. This gap is a direct result of the uniform delay between cortex and hippocampal loop and points to a lack of a potentially important feature in our model.

#### Temporal dispersion

**Figure 7:**
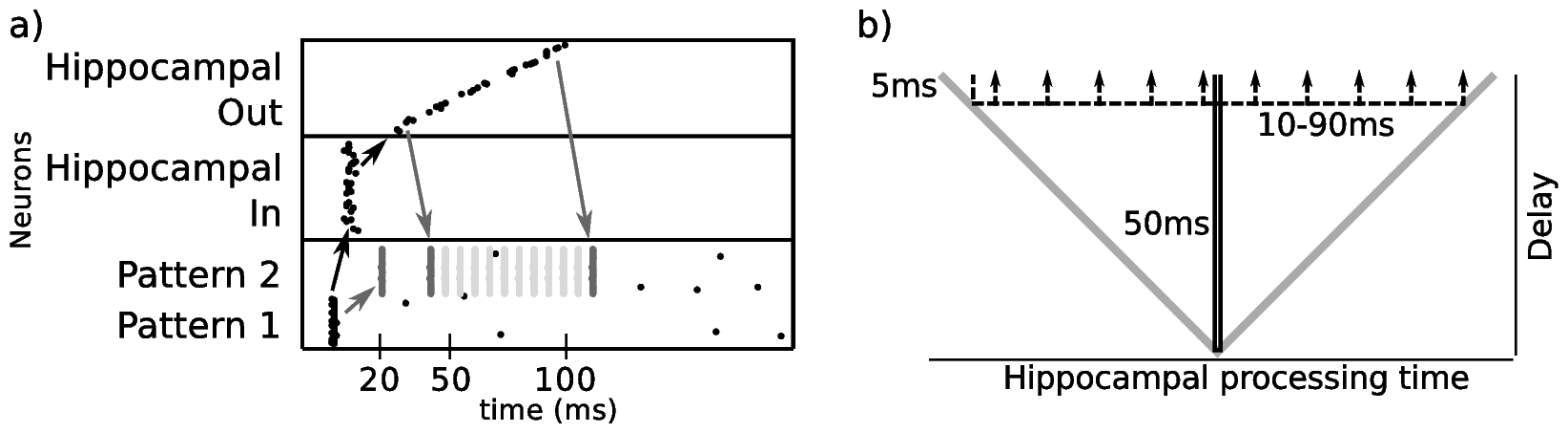
Temporal dispersion in the hippocampal loop. **a**) Temporal dispersion between hippocampal input and output layer generates a sequence of spikes that increases the time span in which cortical patterns can be learned. **b**) Anatomical interpretation of the model. The delay between cortex and hippocampal loop is comparatively short. Hippocampal processing time varies between 10–90 ms.

The hippocampus actually includes several parallel loops of different lengths that simultaneously process and project back to cortex. For example, activity reaching the entorhinal cortex are transmitted through a loop containing the dentate gyrus, CA3 and CA1 but also through a loop comprising CA3 and CA1 and through a loop including CA1 alone [20]. On an abstract level, this means that coincidental cortical spikes are processed along several parallel pathways producing a stream of temporally dispersed output spikes. To examine how temporal dispersion affects learning in our model, we introduced heterogeneous conduction delays between cortex and hippocampal input and output such that simultaneous activity in the cortex generates a spatio-temporal return pattern in the cortex between 12–100 ms (see Methods). An anatomical interpretation of this model is that the hippocampus is spatially close to the cortex resulting in short conduction delays, but contains several processing loops that generate a temporally extended output signal (Fig. 7b). This dispersion fills the gap in temporal separation, in which associations of hippocampal and cortical patterns could not be learned (Figs. 7a and 6, bottom row). Again, variability in the delay between cue and target pattern during learning did not harm the learning procedure as long as the delay did not exceed the maximum distance of 140 ms.

### Temporal Compression and Consolidation

#### Repeated recalls speeds up pattern retrieval in network with temporal dispersion

**Figure 8:**
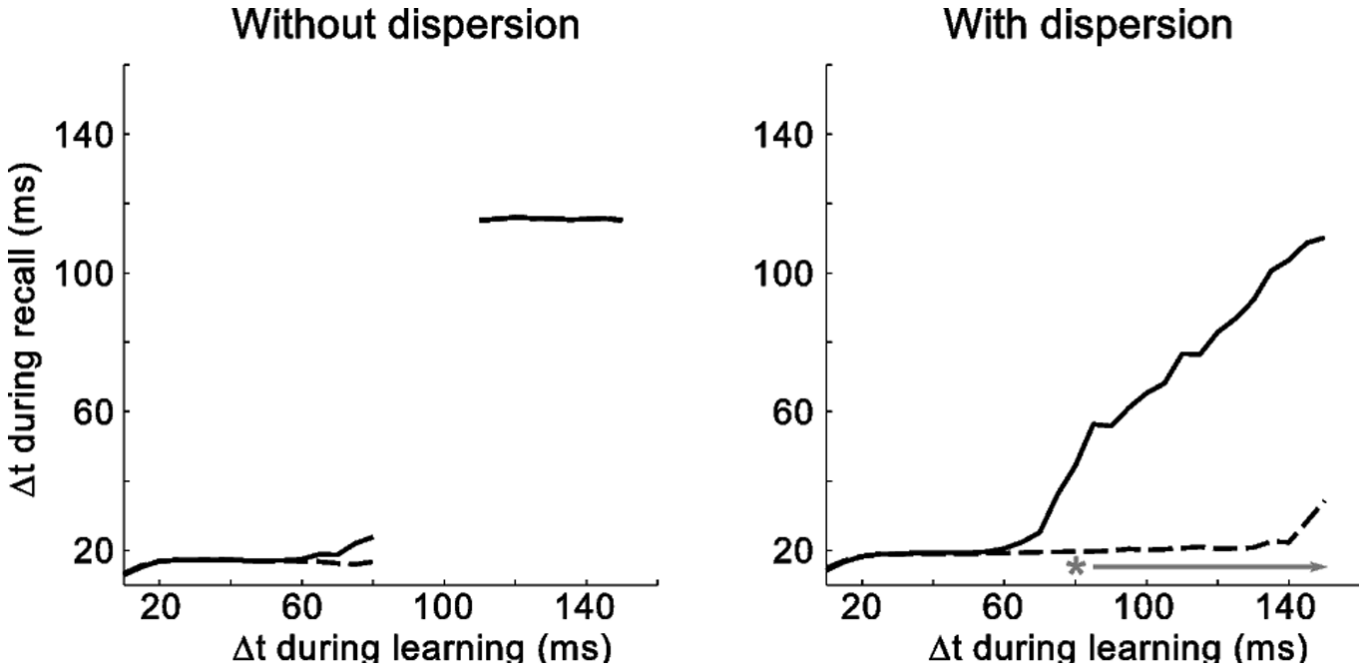
Repeated recalls leads to temporal compression of pattern association. Time separation Δt during recall vs. separation during learning, directly after learning (solid line) and after 300 recalls (dashed line). Each data point represents the average of 10 repetitions in which the median timepoint for the activation of the target pattern was recorded. With temporal dispersion, patterns with any interval of Δt during learning can be learned. When the hippocampal loop is involved in learning (Δt >70), the temporal separation between cue and target pattern directly after learning is result of Δt during learning. After 300 recalls the target pattern is activated after ∼10 ms. In the model without temporal dispersion, the target pattern remains at a temporal separation of either ∼10 ms or ∼120 ms. Asterisk and arrow indicate significant differences between the median timepoint of the target pattern directly after learning and that after 300 recalls (Mann-Whitney-Wilcoxon, p<10^−4^).

Interestingly, the expanded sequence of hippocampal activity enables the model to shorten the temporal separation between two learned patterns and consolidate them in the cortex. To illustrate this behavior, we stored the association between two patterns separated by varying intervals Δ*t* in networks with and without temporal dispersion. After each training session, recall was triggered 300 times and the interval between cue presentation and retrieval was determined. To do so, we computed the median time of all spikes fired by the neurons in the target pattern within a window of 200 ms following the cue. In the network without temporal dispersion, the target pattern is consistently activated after ca. 10 ms for Δ*t* of 10–80 ms and after 120 ms for Δt of 120–150 ms (Fig. 8, left). Note that the temporal separation during retrieval is largely independent of the temporal separation of the stored association and reflects a compression mechanism discussed below. The break in the lines corresponds to the gap in Figure 6, in which no association can be learned by the network. Not surprisingly, the first and the 300th retrieval did not differ in their temporal separation. By contrast, in the network with temporal dispersion, temporal separation during retrieval scales with the temporal separation between the training patterns for Δ*t* >70 ms (Fig. 8, right). The transition point matches the Δ*t*, at which storage begins to depend on the hippocampus. However, after 300 recalls, the second pattern is activated within 10 ms, i.e. directly by cortical neurons. Mann-Whitney-Wilcoxon tests based on the spike-timing directly after learning and after 300 recalls revealed that, for Δt ≥80 ms in the learning phase, the difference becomes significant (p<10^−4^). This process is facilitated by the same compression mechanism coupled with temporal dispersion.

**Figure 9:**
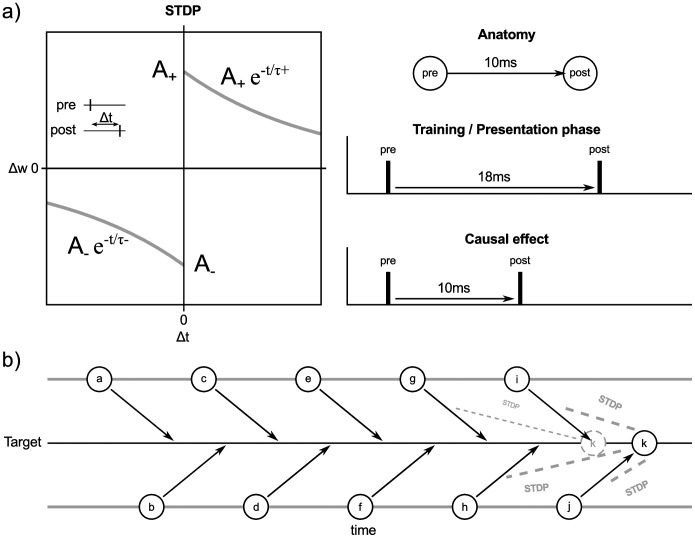
Illustration of the mechanism that compresses the temporal sequence of spikes. **a**) The weight of a connection between a pre- and post-synaptic neuron increases according to the STDP rule, even if the interval between pre- and post-synaptic spike exceeds the delay of the connection. However, when the connection is strong enough, the pre-synaptic neuron can directly cause the post-synaptic neuron to fire. Thereby, the temporal interval of both spikes becomes shortened. **b**) The same effect applies when timed spikes of several neurons are required. In this example, the neurons a-j are connected with neuron k and fire in a temporal sequence. Neuron i and j together can make neuron k spike. However, STDP also increases connection weights from neurons spiking earlier in time (e.g. h and g). After sufficient repetitions, neuron i can directly evoke the spike of the target neuron as the contribution of neuron h already increased the activity level of neuron k. Thereby, neuron k spikes earlier. This mechanism continuous for all preceding neurons.

The compression effect emerges as a side-effect of the STDP rule (Fig. 9). The simplest case of this effect is depicted in Fig. 9a. When a pair of neurons with a synaptic delay of 10 ms is stimulated with a delay of 18 ms between pre- and post-synaptic spiking, STDP still increases the weight of the synapse due to its finite time window. Once a pre-synaptic spike can directly evoke a post-synaptic spike, the delay between both spikes becomes shortened from previously 18 ms during training to 10 ms during recall. The same mechanism can be observed, when joint activity is required to generate post-synaptic spikes. In the network with temporal dispersion, four or more hippocampal spikes from different neurons are required to trigger a spike in a cortical target neuron (Fig. 5). Again, the effect of STDP is strong enough to increase the connection weights of hippocampal neurons that spiked before but did not directly contribute to the post-synaptic spike. After sufficient repetitions, the connections can become strong enough to initiate a spike directly and thereby trigger the post-synaptic spike earlier (Fig. 9b).

#### Repeated recalls leads to consolidation of stored association

**Figure 10:**
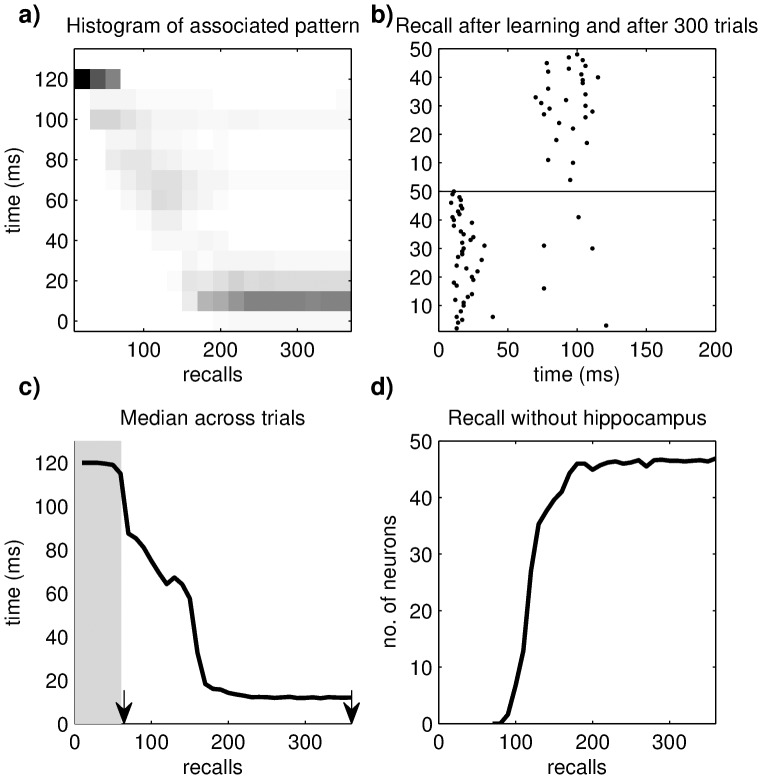
The association between cue and target patterns becomes independent of the hippocampus with repeated recalls. **a**) Histogram of target neuron spikes as a function of time and recalls. At the beginning of the learning phase, neurons in the target pattern fire spikes with a delay of 120 ms. During later learning trials and recall, more and more neurons fire earlier until cortical neurons are directly linked with the target pattern. **b**) Plot of the spikes of the target neurons relative to the onset of the cue pattern. The upper and lower half of the plot depict the spiking pattern directly after learning and after 300 recalls, respectively (indicated by arrows in c). **c**) Median of the histogram in a). Due to the outliers in the recall phase the mean value would not be representative of most spike times. **d**) Number of neurons in the target pattern that can be evoked without the hippocampal loop, as a function of recalls.

That repeated recalls reduce the retrieval separation to 10 ms regardless of the temporal separation during learning indicates that retrieval gradually becomes independent of the hippocampus. We therefore studied this process in more detail in the network with temporal dispersion. As described above, the cue and target patterns are presented to the cortex separated by 120 ms for the first 60 trials, and the target pattern is retrieved earlier and earlier with each subsequent recall (Fig. 10a–c). When retrieval intervals become short enough (<70 ms), STDP can increase the weights of direct connections between cortical neurons in the cue and the target patterns. After around 200 trials the target pattern can be retrieved directly by cortical neurons within ca. 10 ms.

A multitude of experimental studies has shown that lesioning or inactivating the hippocampal formation directly after learning hamper memory performance. However, when the hippocampus is lesioned a few days after training, behavioral performance in a memory task is not affected [34]. We can reproduce this observation in our model, by removing the hippocampus from our network after each trial. To do so, we temporarily set the connection weights from cortex to hippocampus to zero and disabled STDP. We then tested memory performance in this lesioned network by applying the cue pattern and recording the number of spiking neurons in the target pattern. As the retrieval separation analysis suggested, the target pattern could be nearly completely retrieved by the cortex alone after 200 recall trials (Fig. 10d).

### Robustness of Temporal Compression and Consolidation to Parameter Changes

**Figure 11:**
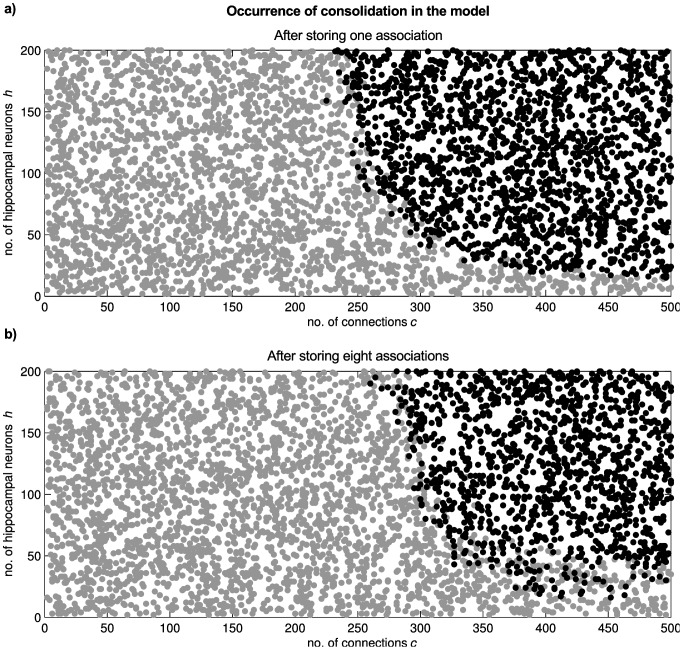
Consolidation across a range of model parameters. Consolidation is robustly observed in our model for a range of two parameters: the number of hippocampal neurons, and the number of cortical cells that each hippocampal cell is connected with. Black dots depict networks that show consolidation, networks represented by circles did not reveal consolidation. Behavior of the model across the parameter space when a) only one or b) eight associations are stored in the network.

At the beginning of the result section, we determined a setting for the parameters of no interest that leads to the desired behavior of the model (e.g. no overloading of the network) and explored for an example setting of the parameters of interest the functional influence on learning. Here, we study how robust the observed compression and consolidation of associations are. We sampled (n = 3000) across the number of hippocampal neurons *h* and the number of input/output connections for hippocampal neurons *c,* and repeated the analyses above (*h* = 100, *c* = 300) for each network. A network is considered to exhibit the consolidation function, if after the first recall the network is not able to recall the target pattern without the hippocampal loop and after 300 recalls the network is able to recall more than 50% of the target pattern without the hippocampal loop. Without further fine-tuning of the parameters of no interest, we found a region in the parameter space in which consolidation was observed (Fig. 11a). In particular, we found that even for models with a much lower number of hippocampal neurons (e.g. h = 20) than the number of cortical cells participating in the stored patterns the reported behavior can be observed. In general, higher values of *h* and *c* do not harm the consolidation process while there exist lower thresholds.

Additionally, we tested the robustness of the model, when the network stores multiple associations. In detail, eight cue patterns were pairwise associated with eight target patterns, as described above. After training, we tested the ability of the network to consolidate the association that was stored first to examine the effect of storing new associations that might potentially interfere. Otherwise, we used the same parameters for our simulations as above. We found that although slightly more connections and hippocampal neurons are necessary, consolidation is similarly reliable even when potentially interfering associations are stored in the cortico-hippocampal network (Fig. 11b).

In summary, the results support the hypothesis that a certain amount of convergence/divergence between cortex and hippocampal loop is needed to facilitate learning and consolidation of associations through the hippocampal loop. However, within certain boundaries the function of the model is robust to changes in the parameters of interest.

## Discussion

In this study, we investigated *in silico* the functional contribution of two anatomical properties that govern the connections between cortex and hippocampus: (i) a high degree of connectivity resulting in strong convergent and divergent projections and (ii) conduction delays resulting from the detached location and the processing time of the hippocampal formation. Our analyses showed, that these network properties give rise to important characteristics of hippocampally-dependent learning. Cortical activities evoke a unique spatio-temporal signature in the hippocampal loop that can be associated with other cortical patterns. Therefore, comparatively few hippocampal neurons are able to unambiguously address cortical patterns. With this mechanism, associations can be learned with temporal delays that are not accessible with STDP in the cortex. Including pathways with varying delays in the cortico-hippocampal network, we found that repeated recalls of stored associations shortens the interval between cue presentation and retrieval of the target pattern until a target pattern can be retrieved directly from cortical neurons. We thus suggest that simple properties of cortico-hippocampal connectivity might provide the basis for the consolidation of episodic memories.

### Model for Consolidation and Relationships to Conceptual Models

The idea of a unifying and species-independent framework of the hippocampus has been proposed by many authors [10], [20], [22], [35]–[38]. Our simulations could be a valuable foundation to bring those theoretical frameworks alive by providing mechanistic explanations for functions associated with the hippocampus and complementing experimental findings. For example, it has been suggested that diverging backprojections from the hippocampus to higher cortical areas are associatively modified during memory formation [39], [40]. We were able to show that unique assignments between cue and target pattern can be established with spatio-temporal patterns in the hippocampal loop that serve as link to cortical information. It is important to note, that the functional correlate of an information is represented by cortical neurons. The converging connections and heterogenous conduction delays between cortex and hippocampal loop transform the patterns into spatio-temporal sequences that act as unique identifier in a small set of hippocampal neurons to facilitate the formation of associations with other cortical information. In a disrupted hippocampal loop only the association but not the cortical information themselves would be affected. This is consistent with experimental results indicating that the hippocampus is involved in learning temporal sequences and bridging temporal gaps [41]–[43].

It has been suggested before that repetitive reactivation of learned associations leads to consolidation [44]. During rapid eye movement (REM) sleep and slow-wave sleep (SWS), hippocampal neurons in rats fire spikes in similar sequential order as during task performance [45], [46]. This so-called replay activity is believed to be important for consolidating memories in the neocortex (for a review, see [47]). Our results suggest that the connectivity properties between cortex and hippocampus and multiple loops within the hippocampus can provide a mechanistic explanation for consolidation.

Our model contrasts with previous models of consolidation [48], [49] in several relevant aspects. First, we incorporate the spatial properties of the cortico-hippocampal network in our model through conduction delays that can be mapped to distances between anatomical regions. The previous models treat the network as a single point. Second, consolidation is not actively controlled by the hippocampus in our model, but emerges from repeated recalls that are triggered by cortical activity. By contrast, in the previous models, the hippocampus (medial temporal lobe, respectively) imposes both cortical cue and target patterns during the consolidation process to imprint the association in the slower changing cortico-cortical connections. Finally, both previous models represent episodic memories as instantaneous patterns of activity, whereas we view them as sequences of activity patterns [20]. Through sequential activity in the hippocampus, temporal separation during retrieval decreases until cortical neurons alone can recall the target pattern. While in the context of synfire chains this effect has been regarded as an unwanted property that can be overcome by a triphasic STDP rule [50], in the context of sequential learning this behavior seems to be functionally important. We suggested previously that consolidation is the process of converting temporally extended sequences of patterns into patterns without much temporal extend, which represent semantic memories stored in the cortex [20].

Several aspects of our model could be tested experimentally. For instance, the spatio-temporal transformation of cortical patterns in the hippocampal loop originates in our model from converging/diverging connections between cortical and hippocampal areas with heterogeneous conduction delays. An experimental assessment of the conduction delays between cortical and hippocampal/parahippocampal areas would provide realistic constrains for our model assumptions. We predict that conduction delays between cortex and hippocampus are heterogeneous, similar to findings within the hippocampus [17].

We observed in our model that associations between patterns separated by larger intervals are initially stored in connections with longer delays, and that with repeated recalls are gradually transferred to connections with shorter delays. If the same principle holds true in the real hippocampus, it is possible that different subareas of the hippocampal formation are involved in consolidation for different amount of time after memory formation. The longer the loop through the subarea, the shorter the duration that it is required for consolidation. This prediction could be tested by selectively lesioning DG, CA3, CA1, and entorhinal cortex at different time points after learning.

Finally, consolidation in our network leads to a gradual compression of the delay between cortical activation and retrieval of the target pattern. If cortical activity could be uniquely assigned to the representation of certain items, then one could measure the time it takes for recall of the target item in animals that were trained on sequential associations of item pairs with different temporal separation.

### Special Features of Our Model

The conduction delays used in our model have been chosen to accommodate the fact that connections from the neocortex to the hippocampus must be longer than within the neocortex. The settings of the neocortical networks are based on a previous study [18]. Given estimates of the distance/latency function in the hippocampus [17] and analyses of conduction delays between entorhinal cortex and dentate gyrus [51] in rats, the delay of 50 ms for one direction does not appear to be unrealistic for rats. However, the major point of this setup is that the spatial distance between neocortex and hippocampus introduces a period of time that facilitates learning of associations where STDP within the cortex has no effect any more. A high degree of convergence and divergence within the loop is a necessary property of the model and is consistent with the biological system. The relevance for bridging latencies in the range of hundreds of milliseconds has been shown, for example, in trace eyeblink conditioning studies [52]–[54].

However, the range of temporal separations of 10–140 ms, for which associations between two pattern can be stored in our model, might appear small in light of other experimental findings. Studies have shown that hippocampal activity increases when a period of several seconds must be bridged [55], [56]. So-called time cells in CA1 encode time on the order of seconds in a similar invariant manner to place cells in the context of navigation [55]. We note that our model is a proof of principle, and does not elaborate on the details of the hippocampus with its complex network of subareas. From an evolutionary perspective, it could be feasible that the complex and specific structures are a result of a continuous elaboration of the hippocampal loop that improved the performance in learning associations and bridging a period of time. It remains an open question whether and how cortical areas are also involved in maintaining the representation of information. For example, in computational simulations it has been shown that the cortical network itself can maintain a representation for several seconds using NMDA-receptors, e.g. [19].

### Limitations and Future Work

We observed that a hippocampal loop with temporal dispersion requires more repetitions to learn an association as fewer neurons are active at the same time. It would have been possible to change the STDP rule to yield faster learning, but as mentioned in the Introduction, we opted for minimal changes to the network to maintain focus on the properties of cortico-hippocampal connectivity. Another consequence of the decreased number of hippocampal neurons processing information is that it becomes more difficult to dissociate two previously learned associations. Therefore increasing temporal dispersion should possibly be accompanied by an increase of the number of neurons. Again, we opted for minimal changes to the network to isolate the effects of temporal dispersion.

It turns out to be difficult to train our model on sequences of more than two patterns. In our model, the retrieved target pattern is spread temporally as compared to the cue pattern. If we were to use the retrieved pattern as a cue to sequentially drive a second target pattern, there would be less activity in the hippocampus in the second step, which makes it more difficult for the hippocampus to drive cortical activity. It is feasible that the biological cortical network performs a clean-up operation at each step of the sequence to facilitate the storage of longer sequences. We did not pursue this possibility further here, because this study focused on the function of the connectivity between cortex and the hippocampal loop and including complex cortical dynamics would confound the results. Future work is needed to reveal the neuronal mechanisms that can account for robust and more complex sequence learning.

Similarly, our model does not include the internal structure of the hippocampus, and therefore does not exhibit other well-known properties and functions of the hippocampal formation, such as for instance place or grid cells [57], [58]. Also, our model stores and retrieves association only in one temporal direction whereas replay activity has been observed in both the forward and reverse direction [59]–[61]. It remains a challenging and interesting task to amend this network by hippocampal subareas such as dentate gyrus, CA3, etc. with their complex intrinsic dynamics [62] to understand how these hippocampal networks interact with the properties of our current model.

### Evolutionary Perspective on the Hippocampus

Since the hippocampal formation was highly conserved in the evolution of mammals [2], [63] while the neocortex underwent large changes, it has been suggested that differing experimental findings might result from the variety of neocortical input rather than from species-specific differences of the hippocampal regions [1], [2]. Our results support this hypothesis. We found, that the functionality of the model is not sensitive to parameter changes. Therefore, the general scheme of the cortico-hippocampal loop (convergence/divergence, spatially distant unit, parallel processing streams) appears to be a design that can be functionally exploited by a variety of species with varying brain sizes and cortical areas to store associations. Therefore, despite species-dependent differences, this common design points to a species-independent function of the hippocampal formation that may underlie differing experimental observations.

Our study could provide a link between form and function of biological networks that is mostly neglected in computational neuroscience. In contrast to most artificial encodings, genes in biological organisms generate gradients of protein concentration [64], [65], which in turn serve as guide for the placement of neurons and their axonal and dendritic growth cones [66]–[68]. The network connectivity properties so important in our model are a direct result of spatial structures [17], [69]. In our opinion, the parameters of our model follow biologically plausible dimensions, such as the distance between two networks and the branching degree of neurons, that may proof useful to connect studies analyzing the impact of genes on the spatial structure of networks with the function that emerges from them.

Computational approaches along these ideas mainly focus on the spatial organization of networks involved in spatial processing, like vision [66], [70], navigation and locomotion [71]–[73]. The study at hand highlights that also sequential problems, like learning temporal associations, can emerge from networks designed in spatial categories. From this perspective, differing conduction delays that directly result from distances between neurons [17], [74], [75], could be a crucial property of neural networks to understand how episodic memories are formed.

## Conclusion

Overall, our results suggest that certain functions previously attributed to the hippocampus (e.g. addressing cortical areas, temporal compression) may arise from the way the hippocampal network is attached to a cortical network. Of course, this does not challenge the fact that the hippocampus contains functionally important subregions necessary for the maintenance and linkage of information. However, our results suggest that the connectivity structure of the cortico-hippocampal network has mechanisms to build and consolidate associations. From this perspective, the high degree of connectivity, the spatial extend of the hippocampal loop (resulting in delays of signal transmission) and the parallel processing ways within the loop might represent universal and functionally meaningful principles of the hippocampus.
